# A Polyzwitterionic@MOF Hydrogel with Exceptionally High Water Vapor Uptake for Efficient Atmospheric Water Harvesting

**DOI:** 10.3390/molecules29081851

**Published:** 2024-04-18

**Authors:** Jian Yan, Wenjia Li, Yingyin Yu, Guangyu Huang, Junjie Peng, Daofei Lv, Xin Chen, Xun Wang, Zewei Liu

**Affiliations:** School of Environment and Chemical Engineering, Foshan University, Foshan 528000, China; yanjian@fosu.edu.cn (J.Y.); liwenjia2024@163.com (W.L.); yuyingyin946@163.com (Y.Y.); hgy2024@163.com (G.H.); cepengjunjie@fosu.edu.cn (J.P.); lvdaofei@163.com (D.L.); chenxin@fosu.edu.cn (X.C.)

**Keywords:** PML hydrogel, MOFs, atmospheric water harvesting, hygroscopic salt

## Abstract

Atmospheric water harvesting (AWH) is considered a promising strategy for sustainable freshwater production in landlocked and arid regions. Hygroscopic salt-based composite sorbents have attracted widespread attention for their water harvesting performance, but suffer from aggregation and leakage issues due to the salting-out effect. In this study, we synthesized a PML hydrogel composite by incorporating zwitterionic hydrogel (PDMAPS) and MIL-101(Cr) as a host for LiCl. The PML hydrogel was characterized using various techniques including X-ray diffraction (XRD), scanning electron microscopy (SEM), Fourier transform infrared (FTIR), and thermogravimetric analysis (TGA). The swelling properties and water vapor adsorption-desorption properties of the PML hydrogel were also assessed. The results demonstrate that the MIL-101(Cr) was uniformly embedded into PDMAP hydrogel, and the PML hydrogel exhibits a swelling ratio of 2.29 due to the salting-in behavior. The PML hydrogel exhibited exceptional water vapor sorption capacity of 0.614 g/g at 298 K, RH = 40% and 1.827 g/g at 298 K, RH = 90%. It reached 80% of its saturated adsorption capacity within 117 and 149 min at 298 K, RH = 30% and 90%, respectively. Additionally, the PML hydrogel showed excellent reversibility in terms of water vapor adsorption after ten consecutive cycles of adsorption-desorption. The remarkable adsorption capacity, favorable adsorption-desorption rate, and regeneration stability make the PML hydrogel a potential candidate for AWH. This polymer-MOF synergistic strategy for immobilization of LiCl in this work offers new insights into designing advanced materials for AWH.

## 1. Introduction

Water resources constitute the fundamental basis for human survival and development, and the issue of water scarcity remains a significant global challenge. According to statistical data from the World Meteorological Organization, as of 2020, over 2 billion people worldwide lacked access to safe drinking water. Projections indicate that by the year 2050, approximately 5 billion people globally will confront challenges related to water scarcity [1,2]. Atmospheric water primarily exists in the form of water vapor and suspended water droplets (mist or clouds), constituting an often overlooked source of freshwater. Estimated to be around 10^18^ L, equivalent to 10% of the global freshwater resources [3], the gaseous water capacity in the atmosphere is a substantial and continuously replenished freshwater source through the global hydrological cycle [4]. Therefore, developing efficient atmospheric water harvesting technologies holds significant research value and practical significance in alleviating regional water scarcity issues. This topic has gained widespread global attention and is recognized as a challenging research subject.

Currently, fog condensation, low-temperature condensation, and adsorption separation technologies are the primary techniques employed for atmospheric water harvesting [5,6,7]. Among these technologies, adsorption separation technology offers several advantages such as a wide working humidity range, low energy consumption, utilization of solar energy for operation, minimal equipment investment, and operational simplicity. Atmospheric water harvesting (AWH), employing adsorption separation as its core mechanism, represents a highly promising water harvesting technology [8,9,10]. The effectiveness of this process is primarily determined by the choice of adsorbent.

Hygroscopic inorganic salts, such as LiCl and CaCl_2_, are cost-effective sorbents with extremely high water harvesting capacities across a wide range of relative humidity (RH) [11,12]. However, challenges arise during the water absorption process, including salt solution leakage, particle crystallization and agglomeration, and excessive energy consumption for regeneration [13,14,15]. To address these issues, a commonly employed approach involves embedding salt into porous materials, where the matrix functions as a container to load deliquescent salts [16,17,18,19]. Nevertheless, widely used porous composites like silica gel [16], zeolites [20,21], carbon nanospheres [22], metal-organic frameworks (MOFs) [23,24], POPs [25,26], and COFs [27,28,29] often exhibit unsatisfactory performances due to their limited pore volume [30].

To further enhance the water vapor adsorption capacity of adsorbent materials, a class of polymer-salt composites has been rapidly developed by incorporating hygroscopic salt into hydrogel matrices. These hydrogels are cross-linked polymer networks with exceptional swelling properties that enable them to absorb significant amounts of water [31]. The most representative materials in this category include PAM [32], SMAG [33], and PNIPAM [34,35,36]. These polymers offer advantages such as designable hydrophilic-hydrophobic structures, high water capacity and density, cyclic stability, and adaptability to a wide range of relative humidity, making them highly suitable for AWH applications. Unfortunately, these hydrogel matrices suffer from the salting-out effect caused by the high content of salt embedded within them. The salting-out effect leads to the undesired aggregation of polymer chains and suppresses the swelling behavior of these hydrogels [37,38], thereby limiting their water solubility and swelling properties which ultimately impact their overall AWH performance [39].

Zwitterionic polymers represent a novel class of materials that possess both cationic and anionic charged moieties on the same side chain maintaining overall charge neutrality [40,41]. Due to the presence of opposite charges, these polymers exhibit distinctive salt-responsive anti-polyelectrolyte effects, wherein salt species can effectively screen self-associate effects between oppositely charged functional groups [39]. Consequently, these polymers demonstrate enhanced solubility at specific salt concentrations compared to pure water, due to the dissociation of the self-associative interaction. This phenomenon is commonly referred to as the salting-in effect [42].

**Figure 1:**
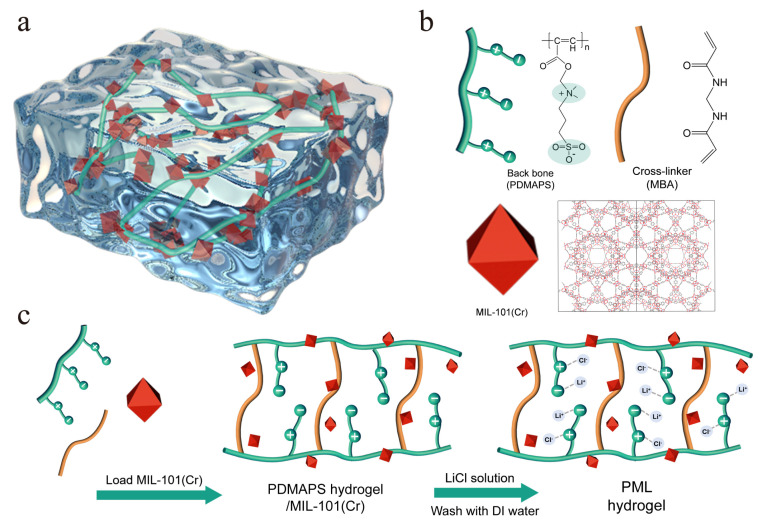
Diagram of (**a**) PML hydrogel. (**b**) Primary components of PML hydrogel. (**c**) Schematic of the fabrication process of PDMAPS, PL, and PML hydrogels.

In summary, we propose that the unique salting-in behavior of zwitterionic polymers facilitates coordination with more salt species and achieves more stretched conformations for moisture capture [43,44]. Incorporating MOFs with high porosity and a large specific surface area into polymers further reinforces salt immobilization and water vapor adsorption [35,36]. In this study, we synthesized a PML hydrogel composite in which zwitterionic poly-[2-(methacryloyloxy)ethyl]-dimethyl-(3-sulfopropyl)ammonium hydroxide (PDMAPS) hydrogel and MIL-101(Cr) together serve as a host for LiCl (Figure 1). We evaluated the enhanced swelling properties and water vapor adsorption-desorption properties of PML hydrogel. Due to the concurrent immobilization of free ions using zwitterionic polymers (PDMAPS) and MOF materials (MIL-101(Cr)), the PML hydrogel exhibited an exceptionally high water vapor sorption capacity of 0.614 g/g at 298 K, RH = 40% and 1.827 g/g at 298 K, RH = 90%. Additionally, the synthesized hydrogel demonstrates remarkable cyclic stability while effectively preventing leakage. The polymer-MOF synergistic strategy for immobilizing LiCl enhances the salting-in effect and subsequently improves water vapor adsorption properties; this approach can provide new insights into designing advanced composite materials for AWH application.

## 2. Results and Discussion

### 2.1. Swelling Properties

**Figure 2:**
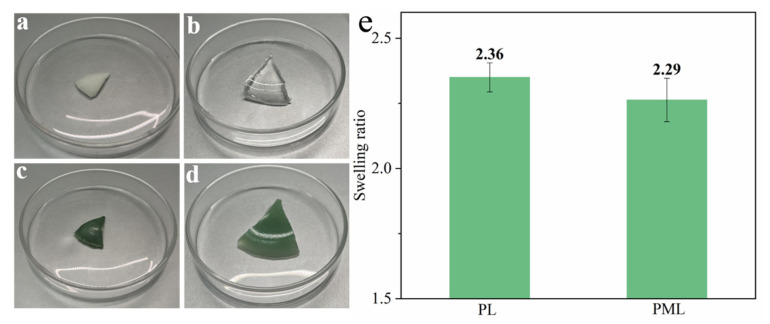
The optical photograph of (**a**) dried PL, (**b**) wet PL, (**c**) dried PML, and (**d**) wet PML hydrogel after swelling in DI water. (**e**) Swelling ratio of PL and PML hydrogels in DI water.

The swelling performance of the hydrogel reflects its water storage capacity. The swelling properties are primarily indicated by the swelling ratio of the hydrogel after swelling in aqueous solution, with a higher swelling ratio indicating a stronger water storage capacity of the material. Figure 2 displays the swelling properties of the synthesized PL and PML hydrogels. From the optical photograph (Figure 2a–d), it can be observed that PL forms a transparent gel, while PML exhibits a uniformly green gel after swelling in DI water. Both PL and PML hydrogels show a significant volume increase, demonstrating excellent swelling performance in the swelling ratio of PL and PML hydrogel swelling. Figure 2e displays the swelling ratio of PL and PML hydrogel in DI water were 2.36 and 2.29, respectively, indicating that the PL and PML hydrogel have good swelling performance and water storage capacity.

### 2.2. XRD Analysis

**Figure 3:**
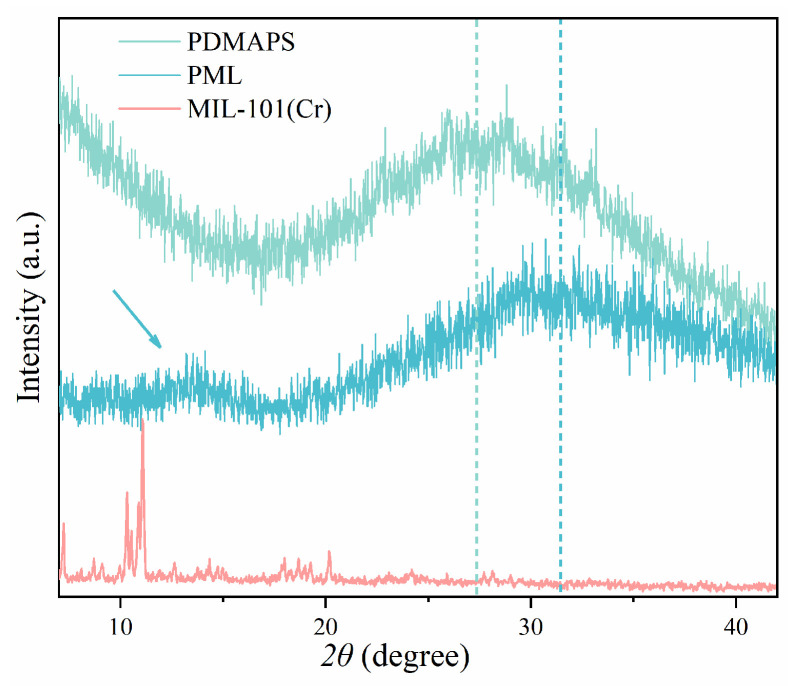
XRD patterns of PDMAPS, PML hydrogels, and MIL-101(Cr).

Figure 3 presents the X-ray diffraction (XRD) pattern of the synthesized PDMAPS, PML hydrogels, and MIL-101(Cr) samples. The XRD pattern of the PDMAPS hydrogel exhibits a broad peak at 2*θ*~27°, indicating its amorphous nature. The XRD pattern of the PML hydrogel did not show any characteristic peaks of LiCl (2*θ*~30°, 35° [40]). Additionally, in the XRD spectrum of PML, the characteristic peak of MIL-101(Cr) at 2*θ*~11° was not found, which may be because the content of MIL-101(Cr) in the PML was low and the amorphous diffraction peak of PDMAPS covers the characteristic peak of the MIL-101(Cr).

### 2.3. SEM Analysis

**Figure 4:**
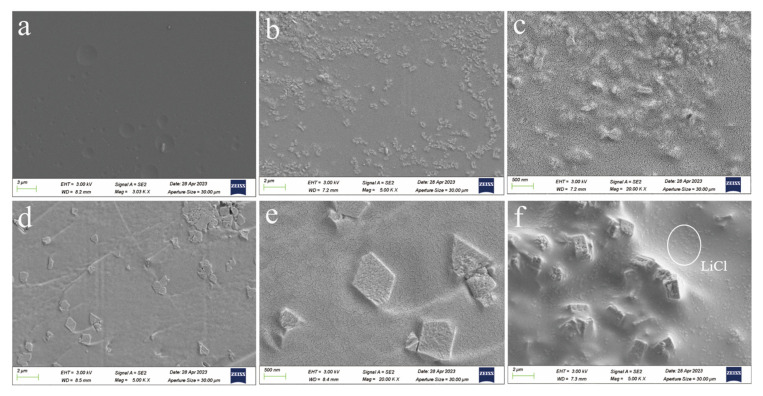
SEM images of (**a**) PDMAPS, (**b**,**c**) PL, (**d**,**e**) PDMAPS/MIL-101, and (**f**) PML hydrogels.

The SEM images in Figure 4 depict the morphologies of PDMAPS, PL, PDMAPS/MIL-101(Cr), and PML hydrogels. It can be observed that the surface of PDMAPS hydrogel (Figure 4a) exhibits a dense and smooth texture, while Figure 4b,c reveals a disordered arrangement of LiCl particles loaded onto the surface of the PDMAP hydrogel. In the case of the PDMAPS/MIL-101(Cr) hydrogel (Figure 4d,e), MIL-101(Cr) particles were dispersed and embedded within the amorphous structure of the PDMAPS, with an average particle size measuring 12.9 ± 1 μm for MIL-101(Cr). Furthermore, Figure 4f demonstrates not only that MIL-101(Cr) was embedded within the PDMAPS, but also LiCl was uniformly loaded onto its surface.

### 2.4. FTIR Analysis

**Figure 5:**
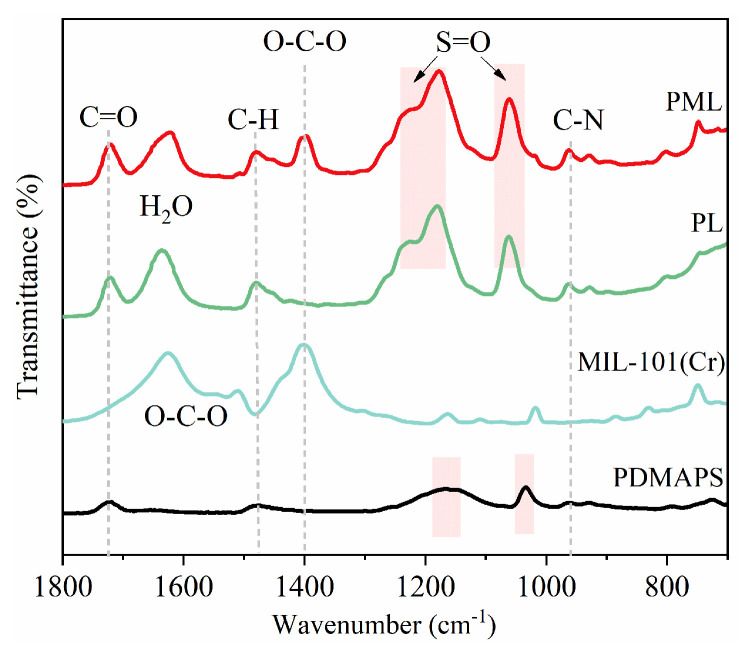
FTIR spectra of PDMAPS, PL, PML hydrogels, and MIL-101(Cr).

The FTIR spectra of the synthesized PDMAPS, PL, PML hydrogels, and MIL-101(Cr) are presented in Figure 5. The characteristic peaks observed in the PDMAPS hydrogel were consistent with those reported in the literature [30]. The peaks at ~1038 cm^−1^ and ~1170 cm^−1^ correspond to the symmetric and asymmetric stretching vibrations of S=O. The peak at ~1480 cm^−1^ represented the C-H stretching vibration of -N^+^(CH_3_)_2_-, while the peak located at ~1723 cm^−1^ was assigned to the stretching vibration of C=O [30]. Upon loading LiCl salt into the PDMAPS hydrogel, significant changes and shifts were observed in the peaks at ~1170 and ~1038 cm^−1^ associated with the stretching vibrations of S=O, indicating that the original interactions between anionic groups (-SO_3_^−^) and cationic (-N^+^(CH_3_)_2_-) in PDMAPS polymer were greatly affected by Li^+^ and Cl^−^. Additionally, the relative strength of the water peak at ~1650 cm^−1^ increased significantly because of the high hygroscopic performance of LiCl. For PML hydrogel embedded with MIL-101(Cr), two new peaks emerged at 1404 and 1625 cm^−1^. The band observed at 1404 cm^−1^ was assigned to the O-C-O symmetrical stretching vibration of terephthalic acid, whereas the band at 1625 cm^−1^ was assigned adsorbed water in the structure. These results further demonstrated that the ions of LiCl salts interacted with the zwitterionic groups of PDMAPS, and MIL-101(Cr) was successfully embedded within the PDMAPS matrix.

### 2.5. Thermogravimetric Analysis

**Figure 6:**
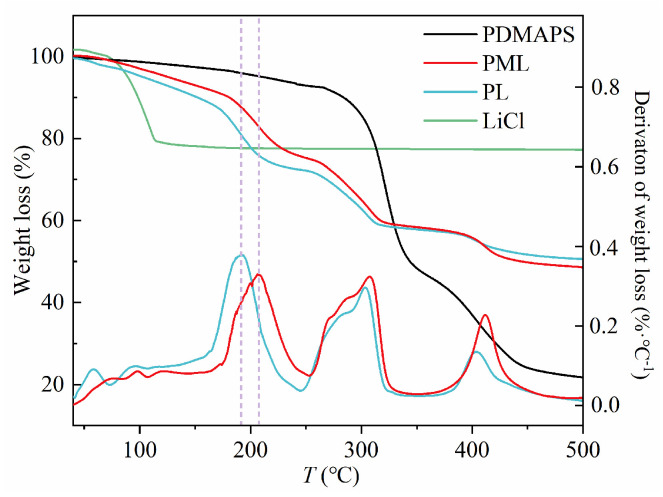
Thermogravimetric analysis of PDMAPS, PL, PML hydrogels, and LiCl.

The TG and DTG spectra of PDMAPS-based hydrogels are presented in Figure 6. For the PDMAP hydrogel, two distinct weight-loss steps were observed: the initial weight loss occurred within the temperature range of 260~350 °C, followed by a second weight loss step at approximately 350~600 °C. In both PL and PML samples, two weight-loss steps and three prominent DTG peaks were identified: the first weight loss step was observed within the temperature range of 160~240 °C, which can be attributed to the evaporation of free and bound water molecules. The second weight loss step took place around 245~340 °C, possibly indicating decomposition of the PDMAPS structure. The final weight loss step occurred between temperatures of 380 and 460 °C when complete degradation of the polymer as well as decomposition of benzenedicarboxylic acid linkers in MIL-101(Cr) took place. Notably, although TG and DTG curves for PL and PML exhibited similarities suggesting comparable thermal stability between these samples, there was a noticeable difference in their respective DTG curves. Specifically for PML, there was a shift in peak temperature from 191 °C to 208 °C after embedding MIL-101(Cr) into the PDMAPS hydrogel matrix. This shift indicates that PML exhibits enhanced interaction with water molecules due to loaded MIL-101(Cr), thereby implying improved capability for capturing water vapor.

### 2.6. Adsorption Isotherms of Water Vapor on the PL with Different LiCl Content

**Figure 7:**
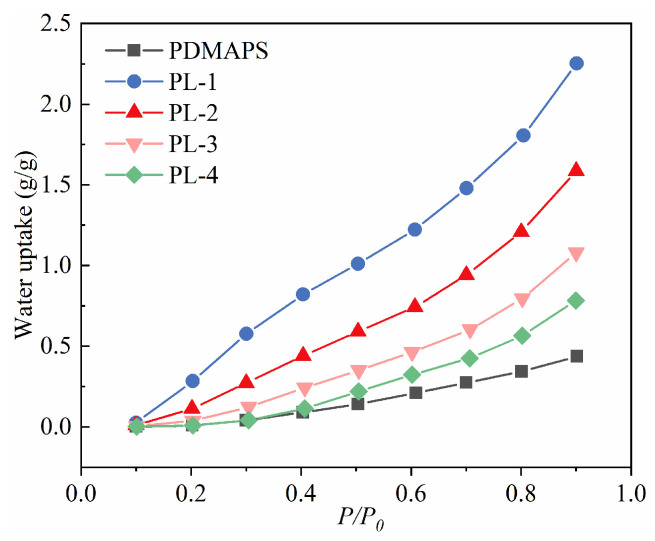
Water vapor adsorption isotherms at 298 K on PL hydrogel with different LiCl content.

The water vapor adsorption isotherms of PDMAPS and PL hydrogels at 298 K are presented in Figure 7. It can be observed that both adsorption isotherms exhibit type III adsorption behavior. The saturated adsorption capacity of PDMAPS is relatively small, reaching 0.087 g/g and 0.436 g/g at RH = 40% and 90%, respectively. Upon loading LiCl into PDMAPS, the saturated adsorption capacity of the hydrogels significantly increases. Specifically, PDMAPS/LiCl-1 achieves a water vapor adsorption capacity of 2.25 g/g at RH = 90%, primarily due to the strong hygroscopicity of LiCl and the excellent water swelling properties of PDMAPS, which synergistically enhance the water vapor adsorption capacity of the composite hydrogels. As the washing time for PL in DI water prolongs, the content of LiCl in the hydrogels continuously decreases, leading to a reduction in their water vapor saturation adsorption capacity. This indicates that loosely bound LiCl is continuously washed out. It is worth noting that precipitated LiCl crystals are observed after adsorption-desorption cycles in prepared PL-2 hydrogel (Figure S2 in Supplementary Materials), indicating that the loosely bound LiCl residues remaining in the PDMAPS structure cannot be completely washed out when the cleaning time was less than 0.5 h. Among non-precipitated LiCl crystals in PL hydrogels, PL-3 exhibits the highest water vapor adsorption capacity with a value of 0.236 g/g at RH = 40% and a saturated value reaching 1.084 g/g at RH = 90%.

### 2.7. Adsorption Isotherms of Water Vapor on the PDMAPS, PL, PML Hydrogels, and MIL-101(Cr)/LiCl

**Figure 8:**
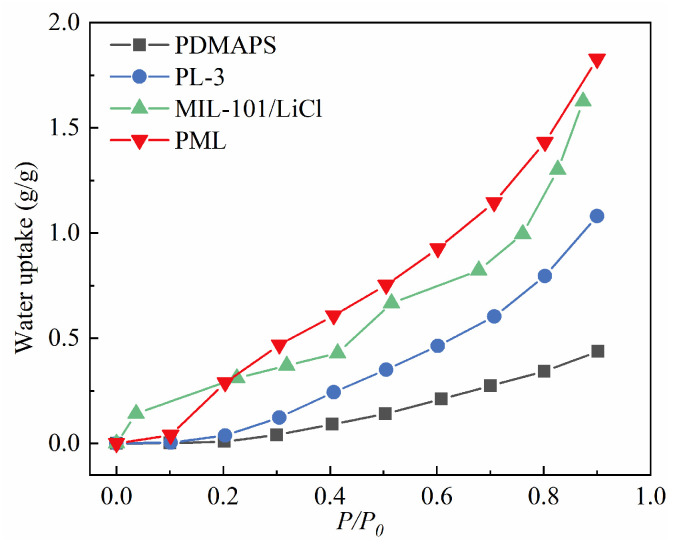
Adsorption isotherms of water vapor on PDMAPS, PL, PML hydrogels, and MIL-101(Cr)/LiCl at 298 K.

The water vapor adsorption curves of PDMAPS, PL, PML hydrogels, and MIL-101(Cr)/LiCl at 298 K are depicted in Figure 8. It can be observed that the water vapor adsorption isotherm of MIL-101(Cr)/LiCl exhibited a stepwise increase, mainly due to the co-existence of the water vapor adsorption behavior of MIL-101(Cr) (Type S) [45] and the multi-step three-phase sorption processes of solid salt (chemisorption, solid-liquid deliquescence, and liquid solution absorption [24]). The saturated adsorption capacity of MIL-101(Cr)/LiCl reached 1.624 g/g, making it one of the solid adsorbents with the highest reported water vapor adsorption capacity to date. However, it should be noted that, although the nanoscale pores of MIL-101(Cr) provided enough space for water storage and induced nanoscale LiCl crystals with fast water sorption/desorption kinetics [24], when LiCl was in excess, there is a tendency for LiCl leakage in the MIL-101(Cr)/LiCl composite material. After the adsorption process, significant dampness and agglomeration are observed in powdered MIL-101(Cr) samples which affects their practical applications.

PML hydrogel exhibited a more pronounced type III adsorption isotherm, and its water vapor adsorption gradually increased with the rise in relative humidity. When RH > 20%, the water vapor adsorption capacity of PML was significantly higher compared to MIL-101(Cr)/LiCl. At RH = 40% and 90%, the water vapor adsorption capacities of PML reached 0.614 and 1.827 g/g, respectively. This represented a 27% and 13% increase compared to MIL-101(Cr)/LiCl. Moreover, it surpassed that of both PDMAPS and PL hydrogels. The enhancement in water vapor adsorption capacity of the PML can be attributed to the synergistic effect of the strong water absorption capacity of MIL-101(Cr)/LiCl and the excellent water swelling properties of PDMAPS. It is worth noting that, compared to the MIL-101(Cr)/LiCl, no significant LiCl crystal precipitation or material dampening was observed in multiple adsorption-desorption cycles of the synthesized PML. This characteristic makes it more favorable for practical AWH applications.

### 2.8. Adsorption Kinetics of Water Vapor on the PDMAPS, PL, PML Hydrogels

**Figure 9:**
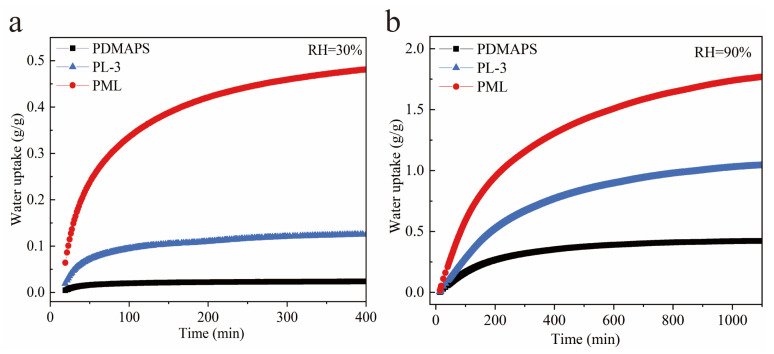
Adsorption kinetics of water vapor on the PDMAPS, PL, PML hydrogels at 298 K, RH = 30% (**a**) and 90% (**b**).

In addition to considering the adsorption capacity, it is crucial to take into account the adsorption kinetics of water vapor when evaluating the practical applications of an adsorbent. The water vapor adsorption kinetic curves of the PDMAPS, PL, and PML hydrogels at 298 K, RH = 30%, and 90% are presented in Figure 9. The PDMAPS hydrogel achieved approximately 80% of its saturated adsorption capacity within 87 min at RH = 30%, and within 370 min at RH = 90%. Compared to the PDMAPS and PL hydrogels, the PML hydrogel has a higher adsorption capacity at RH = 30% and 90%, reaching 80% of its saturated adsorption capacity within 153 min at RH = 30%, and within 530 min at RH = 90%.

**Figure 10:**
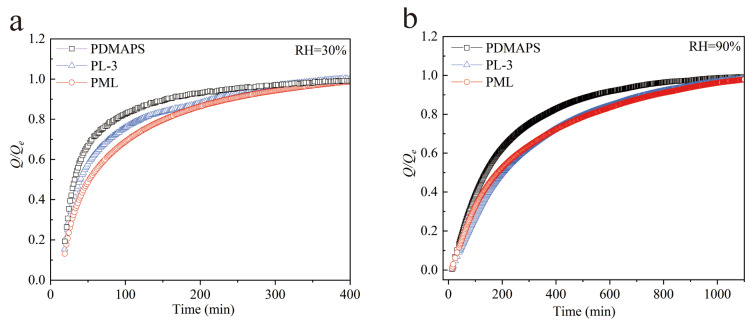
Kinetic curves of fractional transient uptakes of water vapor on the PDMAPS, PL, PML hydrogels at 298 K, RH = 30% (**a**) and 90% (**b**).

The kinetic curves of fractional transient uptakes *Q_t_/Q_e_* of water vapor at different relative humidities are depicted in Figure 10. It was observed that the adsorption rates of water vapor on both the PL and PML hydrogels exhibited comparable behavior. Notably, at low humidity (RH = 30%), the adsorption rate of PL was slightly higher than that of PML, whereas at high humidity (RH = 90%), it was marginally lower compared to PML hydrogel.

This discrepancy can be attributed to the dominant influence of multi-step three-phase sorption processes involving LiCl salt during low humidity conditions [24]. Under high humidity conditions, the water vapor adsorption process of MIL-101(Cr) was significantly enhanced while the water vapor adsorption rate of LiCl was notably lower compared to most solid adsorbents.

### 2.9. Desorption Kinetics of Water Vapor on the PML Hydrogel

**Figure 11:**
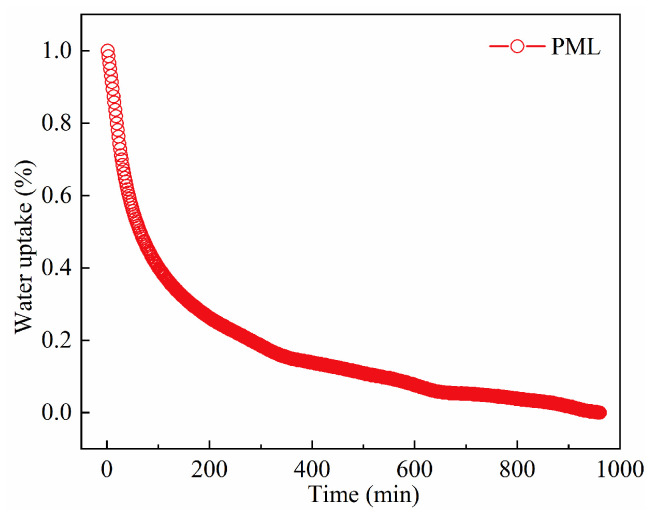
Desorption kinetics of water vapor on the PML hydrogel at 353 K, RH = 0%.

Figure 11 illustrates the desorption kinetics of water vapor on the PML hydrogel at RH = 0% and 353 K, simulating conditions similar to solar-driven atmospheric water harvesting devices in arid desert regions. From the graph, it can be observed that PML hydrogel can lose 80% of its saturated adsorption capacity within approximately 200 min. The residual 20% water takes more than 13 h to completely desorb, and the slow desorption rate is primarily due to the LiCl deliquescence/crystallization process simultaneously occurring with chemical sorption/desorption reactions, which significantly hampers its desorption kinetics [24].

### 2.10. Multiple Adsorption-Desorption Cycles of Water Vapor on the PML Hydrogel

**Figure 12:**
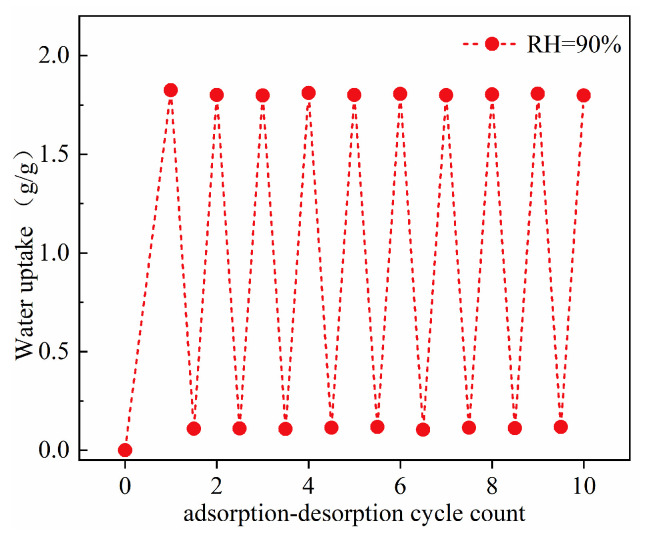
Water vapor adsorption-desorption cycles on the PML hydrogel at 298 K, RH = 90% (adsorption) and 353 K, RH = 0% (desorption).

Regenerability stands out as a pivotal metric for assessing the efficacy of an adsorbent material. Figure 12 presents ten consecutive cycles of water vapor adsorption-desorption on PML hydrogel. Adsorption was executed at 298 K, RH = 90%, and desorption was performed at 353 K, RH = 0%. The maximal equilibrium quantity of water vapor adsorbed by the PML hydrogel reached approximately 1.8 g/g, demonstrating the reversible nature of water vapor adsorption. Moreover, the desorption efficiencies of water vapor from the PML hydrogel exhibited an impressive 93.8%. Importantly, there was no discernible decline in adsorption capacities after undergoing ten consecutive cycles of adsorption-desorption. These findings suggested that under the tested conditions, the PML hydrogel possesses excellent reversibility of water vapor adsorption. These properties could make the PML hydrogel a compelling candidate for deployment as an adsorbent material in applications related to AWH.

## 3. Materials and Methods

### 3.1. Synthesis

Materials. The chemicals utilized in this study were purchased from reputable commercial sources and used without further purification. [2-(Methacryloyloxy)ethyl]dimethyl-(3-sulfopropyl)ammonium hydroxide (DMAPS monomer, 98%), N,N′-Methylenebis(acrylamide) (MBA, 99%), potassium persulfate (KPS, 99.9%), N,N,N′,N′-Tetramethylethylenediamine (TEMED, 99%) nonahydrate, and lithium chloride (LiCl, 99%) were all purchased from Macklin, ShangHai, China. Chromium (III) nitrate (Cr(NO_3_)_3_·9H_2_O, 99%) and terephthalic acid (H_2_BDC) were acquired from Aladdin, ShangHai, China.

PDMAPS hydrogel was synthesized via the free-radical polymerization method following a literature procedure [31,41]. In brief, 2 g of DMAPS monomer was mixed with 0.02 g initiator KPS (1 wt%) and 0.01 g cross-linker MBAA. Then, the mixture was dissolved in 5 mL DI water. Afterward, to purge the mixture with nitrogen gas to eradicate the dissolved oxygen, stirring continued for 10 min. Next, 20 μL of the crosslinker accelerator TEMED was finally added to the mixture, stirring continued for 1 min. The mixture was left to react at room temperature for 12 h. PDMAPS hydrogel was obtained after washing with DI water. Finally, PDMAPS hydrogel was dried in an oven at 80 °C to obtain the dried PDMAPS with reduced volume.

PL hydrogels were fabricated by immersing the PDMAPS hydrogel into LiCl salt aqueous solutions (4 mol/L) for 12 h. After all of the LiCl solution was absorbed by the hydrogel, the polymer hydrogel was soaked separately in DI water (15 min, 0.5 h, 1 h, 2 h) to clean the remaining LiCl on the surface of PDMAPS hydrogel. Finally, PDMAPS/LiCl hydrogels (corresponding named PL-1, PL-2, PL-3, PL-4) with different mass ratios of LiCl to PDMAPS were obtained. The PL hydrogels were dried at 80 °C before further use.

MIL-101(Cr) and MIL-101(Cr)/LiCl. The MIL-101(Cr) was synthesized by hydrothermal method following a literature procedure [46]. MIL-101(Cr)/LiCl was obtained by post-synthesis method following a literature procedure [18]. In brief, a specified quality of LiCl (3 mg/mL) was poured into a pre-dispersed MIL-101(Cr) aqueous suspension (5 mg/mL). Then, the mixture was stirred overnight to let Li^+^ ions and Cl^-^ ions infiltrate into the pores of MIL-101(Cr). Afterward, the obtained composite sorbent was fast-washed with ethanol to remove the remaining LiCl solution adhered to the external surface of MOFs. At last, the LiCl@MIL-101(Cr) was activated by heating at 65 °C for 24 h and dehydrating at 100 °C for 2 h.

PML hydrogel was synthesized with the otherwise same procedure as PL hydrogels. For the PML hydrogel, 2 g of DMAPS monomer was mixed with 0.02 g KPS (1 wt%) and 0.01 g MBAA first. Then, the mixture was dissolved in 5 mL DI water, and 0.15 g MIL-101(Cr) was added to the mixture. After stirring for 10 min, 20 μL of TEMED was added to the mixture, and further stirred for 1 min. Next, the mixture was left to react at room temperature for 12 h. Afterward, PDMAPS/MIL-101(Cr) hydrogel was obtained after washing with DI water. Then, the PDMAPS/MIL-101(Cr) hydrogel was immersed in LiCl salt aqueous solution (4 mol/L) for 12 h and soaked in DI water for 1 h continuously. Finally, the prepared PML hydrogel was dried in an oven at 80 °C to obtain the dried PDMAPS with reduced volume.

### 3.2. Characterization

X-ray diffraction patterns were obtained using a Rigaku D/M-2200T automated diffraction system (Ultima IV, Rigaku, Tokyo, Japan) equipped with Cu-Kα radiation (λ = 1.5406 Å). The measurements were carried out at room temperature, employing a 2*θ* range of 3–80°, a scan speed of 2°/min, an operating voltage of 40 kV, and a current of 40 mA. Scanning electron microscopy (SEM) analysis was performed using a ZEISS Gemini 300 instrument after the deposition of a thin layer of gold. Thermogravimetric analyses were performed using a TA Q5000 analyzer (TA instruments, New Castle, DE, USA), which ramped the temperature from ambient to 700 °C at a heating rate of 10 °C/min under a continuous nitrogen gas atmosphere. Fourier transform infrared (FTIR) spectra were measured by Thermo Niolet iN10 instrument (Thermo Fisher Scientific, Waltham, MA, USA). The test spectral resolution was 4 cm^−1^, the recorded range was 400/600–4000 cm^−1^, and the number of scans was 32. Before the measurements, the sample underwent an initial outgassing process overnight under vacuum at 393 K.

### 3.3. Adsorption of Water Vapor

A multiple-station gravimetric vapor sorption analyzer (BSD-VVS, BSD instrument, Beijing, China) was used to measure the water vapor isotherms, adsorption/desorption kinetics, and the adsorption capacity in multiple water vapor adsorption-desorption cycles. Before measurement, the samples were activated at 150 °C for 10 h. The dry nitrogen gas and the water vapor gas were mixed and then heated to the setting temperature. The relative humidity (RH) was precisely controlled and monitored by a humidity probe. The real-time weight change of the samples was measured by a microbalance with an accuracy of 1 μg. Adsorption was considered to reach the kinetic equilibrium when the change of sample mass was lower than 0.002 mass%/min. The water vapor isotherms were obtained by measuring a series of equilibrium water vapor uptake at different relative humidities. The adsorption kinetics curves were plotted by measuring the real-time weight change of the samples at fixed conditions.

### 3.4. Calculation of the Swelling Ratio

The swelling properties of hydrogels are primarily indicated by the swelling ratio. The swelling ratio of hydrogels in DI water was calculated using the following Equation:

$$Swelling\;ratio=\frac{m_{1}-m_{0}}{m_{0}}$$

where *m*_0_ (g) is the original weight of the dried hydrogel, *m*_1_ (g) is the weight of hydrogel after immersing in DI water for 12 h.

## 4. Conclusions

In this study, we synthesized a composite hydrogel consisting of zwitterionic hydrogel, MOFs, and hygroscopic salt to create a PML hydrogel. Due to the concurrent immobilization of free ions using PDMAPS and MIL-101(Cr), the PML hydrogel exhibits an enhanced and attractive high water vapor sorption capacity of 0.614 g/g at 298 K, RH = 40% and 1.827 g/g at 298 K, RH = 90%. Notably, the PML hydrogel demonstrates a higher adsorption capacity at RH = 90%, reaching 80% of its saturated adsorption capacity in 149 min. Moreover, the synthesized PML hydrogel exhibits exceptional cyclic stability and effectively prevents leakage. This class of polymer-MOF hydrogels along with the synergistic strategy for LiCl immobilization hold great potential for advancing AWH applications while providing valuable insights for designing AWH materials with improved adsorption performance and stability.

## Data Availability

Data are contained within the article and Supplementary Materials.

## Supplementary Materials

The following supporting information can be downloaded at: https://www.mdpi.com/article/10.3390/molecules29081851/s1, Figure S1: Water vapor adsorption isotherms on pure LiCl, PL-3 and PML hydrogels at 298 K. Figure S2: Optical photographs of (a) PL-2 and (b) PL-3 hydrogels after one adsorption-desorption cycle. Figure S3. Thermogravimetric curves of PML hydrogel before and after an adsorption-desorption cycle. Figure S4. FTIR spectra of PML hydrogel before and after an adsorption-desorption cycle. Figure S5. N_2_ adsorption isotherm of MIL-101(Cr) at 298 K. Table S1. Pore structure parameters of MIL-101 (Cr). Figure S6. Water vapor adsorption isotherm of MIL-101(Cr) at 298 K. Table S2. Comparison of the swelling ratio of the hydrogels for atmospheric water harvesting in the literature. Table S3. Comparison of the water capacity of sorbents in the literature [47,48,49].
