# Supplementary material for: A Polyzwitterionic@MOF Hydrogel with Exceptionally High Water Vapor Uptake for Efficient Atmospheric Water Harvesting
Source: Molecules. 2024 Apr 18;29(8):1851. doi: 10.3390/molecules29081851 (PMC11054390; doi:10.3390/molecules29081851)
Supplement: Supplementary file 1 [file molecules-29-01851-s001.zip › molecules-2875853-supplementary.pdf]

Supporting information (SI)

A Polyzwitterionic@MOF Hydrogel with Exceptionally High Water Vapor Uptake for Efficient Atmospheric Water Harvesting

Jian Yan, Wenjia Li, Yingyin Yu, Guangyu Huang, Junjie Peng, Daofei Lv, Xin Chen, Xun Wang * and Zewei Liu *

School of Environment and Chemical Engineering, Foshan University, Foshan 528000, China; yanjian@fosu.edu.cn (J.Y.); liwenjia2024@163.com (W.L.); yuyingyin946@163.com (Y.Y.); hgy2024@163.com (G.H.); cepengjunjie@fosu.edu.cn (J.P.); lvdaofei@163.com (D.L.); chenxin@fosu.edu.cn (X.C.)

* Correspondence: cexunwang@fosu.edu.cn (X.W.); liuzewei@fosu.edu.cn (Z.L.)

Contents

S1. Water vapor adsorption isotherms on pure LiCl, PL-3 and PML hydrogels

S2. Optical photographs of PL hydrogels after adsorption-desorption cycle

S3. Thermogravimetric curves of PML hydrogel before and after adsorption-desorption cycle

S4. N_2 adsorption test and water adsorption isotherm of MIL-101(Cr)

S5. Comparison of the swelling ratio of the hydrogels and other materials

S6. Comparison of the water capacity of the hydrogels and other materials

S1. Water vapor adsorption isotherms on pure LiCl, PL-3 and PML hydrogels

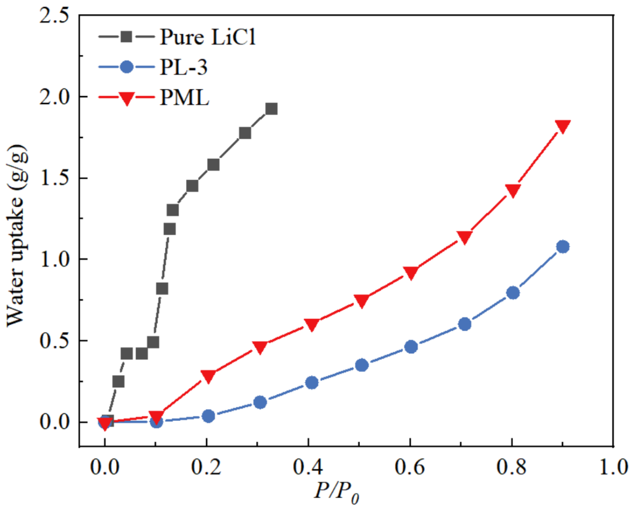

Figure S1. Water vapor adsorption isotherms on pure LiCl, PL-3 and PML hydrogels at 298 K.

S2. Optical photographs of PL hydrogels after adsorption-desorption cycle

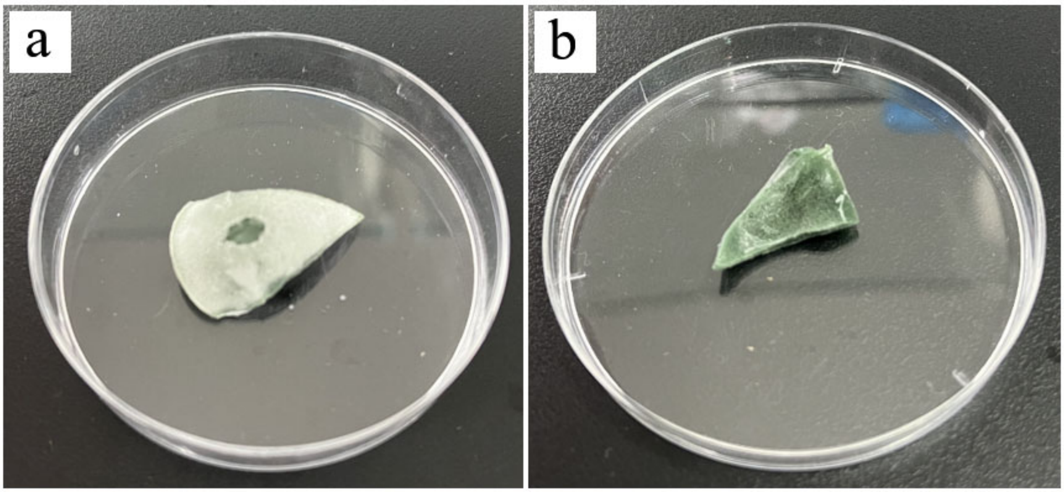

Figure S2. Optical photographs of (a) PL-2 and (b) PL-3 hydrogels after one adsorption-desorption cycle

S3. Thermogravimetric and FTIR curves of PML hydrogel before and after adsorption-desorption cycle

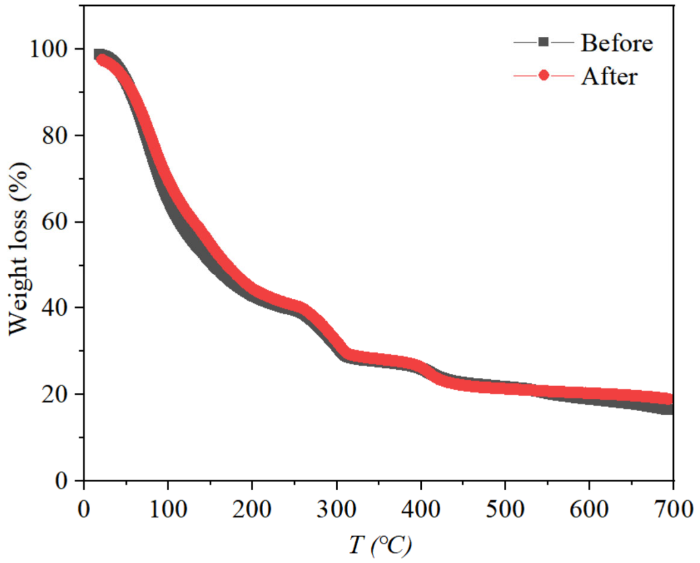

Figure S3. Thermogravimetric curves of PML hydrogel before and after an adsorption-desorption cycle

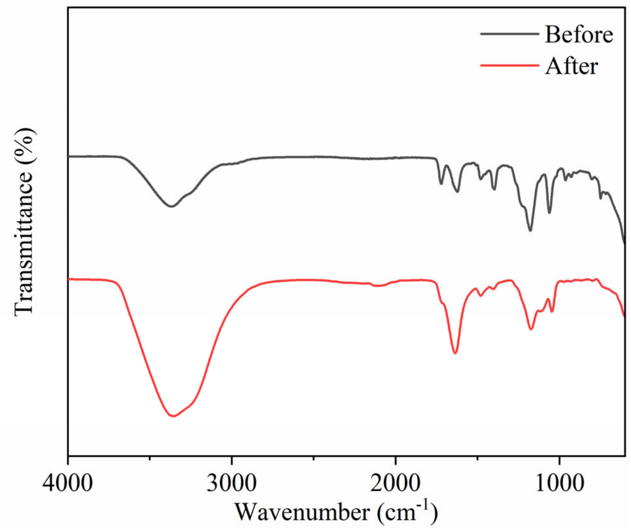

Figure S4. FTIR spectra of PML hydrogel before and after an adsorption-desorption cycle

S4. N_2 adsorption test and water adsorption isotherm of MIL-101(Cr)

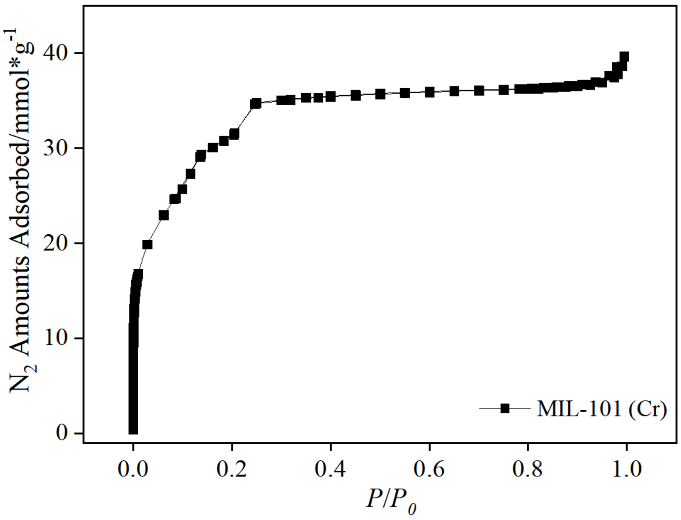

Figure S5. N_2 adsorption isotherm of MIL-101(Cr) at 298 K

Table S1. Pore structure parameters of MIL-101 (Cr)

Sample	BET (m^2/g)	Langmuir (m^2/g)	Pore Volume (cm^3/g)
MIL-101(Cr)	2602	3711	1.25

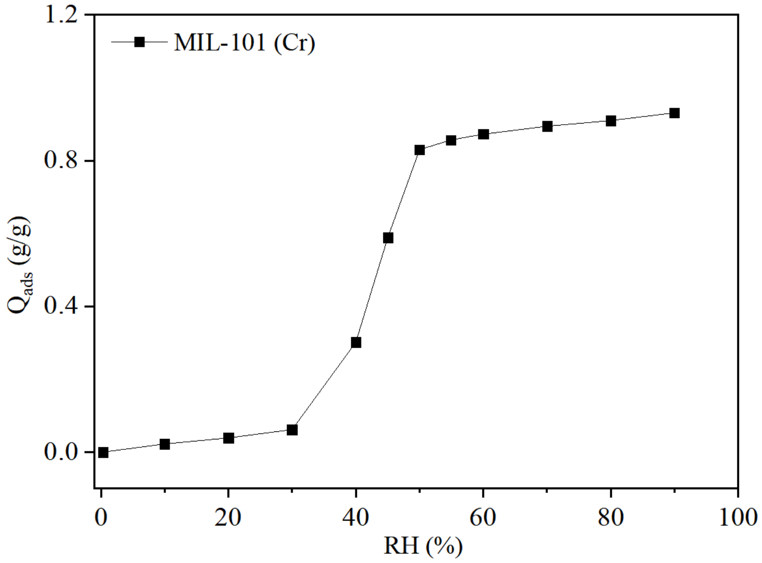

Figure S6. Water vapor adsorption isotherm of MIL-101(Cr) at 298 K

S5. Comparison of the swelling ratio of the hydrogels and other materials

Table S2. Comparison of the swelling ratio of the hydrogels for atmospheric water harvesting in the literature

Materials	Swelling ratio (g/g)	Swelling condition	Reference
PML	1.82	90% RH moisture	**This study**
PML	2.29	DI water	**This study**
PML	9.03	4 mol/L LiCl solution	**This study**
POG	1.54	90% RH moisture	[1]
SMAG	6.3	90% RH moisture	[2]
PAM-CNT	1.75	80% RH moisture	[3]

S6. Comparison of the water capacity of the hydrogels and other materials

Table S3. Comparison of the water capacity of sorbents in the literature.

Materials	Water vapor capacity (g/g) at RH=90%, 25°C	Water vapor capacity (g/g) at RH=30%, 25°C	Reference
PML	1.82	0.43	**This study**
MOF-801	0.28	0.47	[4]
MIL-101(Cr)	1.52[a]	0.25	[5]
PNIPAM	0.21	0.03	[6]
PNIPAM@MIL-101(Cr)	3.6	0.71	[7]
PNIPAM-PPy-Cl (SMAG)	6.5	0.70	[2]
PAM–CNT–$CaCl_2$	1.75[b]	0.68[c]	[8]
SHCP-10 (POPs)	0.76	0.21	[9]
2D ep-POP	0.40	0.1	[10]
COF-432	0.31	0.025	[11]
COFs-480-hydrazide	0.47	0.33	[12]

[a]: $T_{ads/des}$=30°C;

[b]: RH=80%;

[c]: RH=35%

1. Ni, F.; Qiu, N.; Xiao, P.; Zhang, C.; Jian, Y.; Liang, Y.; Xie, W.; Yan, L.; Chen, T. Tillandsia-Inspired hygroscopic photothermal organogels for efficient atmospheric water harvesting. *Angew. Chem. Int. Ed.* **2020,** *59,* 19237–19246.
2. Zhao, F.; Zhou, X.; Liu, Y.; Shi, Y.; Dai, Y.; Yu, G. Super Moisture−absorbent gels for all−weather atmospheric water harvesting. *Adv. Mater.* **2019**, *31*, 1806446.
3. Li, R.; Shi, Y.; Alsaedi, M.; Wu, M.; Shi, L.; Wang, P. Hybrid hydrogel with high water vapor harvesting capacity for deployable solar−driven atmospheric water generator. *Environ. Sci. Technol.* **2018**, *52*, 11367−11377.
4. Furukawa, H,; Gándara, F.; Zhang, Y.; Jiang, J.; Queen, W.; Hudson, M.; Yaghi, O.M. Water adsorption in porous metal–organic frameworks and related materials. *J. Am. Chem. Soc*. **2014**, *136*, 4369–4381.
5. Seo, Y.; Yoon, J.; Férey, Gérard. Porous Materials: Energy-Efficient Dehumidification over Hierachically Porous Metal–Organic Frameworks as Advanced Water Adsorbents. *Adv. Mater*. **2012**, *24*, 806–810.
6. Karmakar, A.; Mileo, P.G.M.; Bok, I.; Peh, S.B.; Zhang, J.; Yuan, H.; Maurin, G.; Zhao, D. thermo-responsive MOF/polymer composites for temperature-mediated water capture and release. *Angew. Chem. Int. Ed.* **2020**, *59*, 11003−11009.
7. Yilmaz, G.; Meng, F.L.; Lu, W.; Abed, J.; Peh, C.K.N.; Gao, M.; Sargent, E.H.; Ho, G.W. Autonomous atmospheric water seeping MOF matrix. *Sci. Adv.* **2020**, *6*, 8605.
8. Li, R.; Shi, Y.; Alsaedi, M.; Wu, M.; Shi, L.; Wang, P. Hybrid hydrogel with high water vapor harvesting capacity for deployable solar−driven atmospheric water generator. *Environ. Sci. Technol.* **2018**, *52,* 11367−11377.
9. Schweng, P.; Mayer, F.; Galehdari, D.; Weiland, K.; Woodward, R. A Robust and Low−Cost Sulfonated Hypercrosslinked Polymer for Atmospheric Water Harvesting. *Small.* **2023**, *19*, 2304562.
10. Byun, Y.; Coskun, A. Epoxy−Functionalized Porous Organic Polymers via the Diels−Alder Cycloaddition Reaction for Atmospheric Water Capture. *Angew. Chem. Int. Ed.* **2018**, *57*, 3173−3177.
11. Nguyen, H.; Hanikel, N.; Lyle, S.; Zhu, C.; Proserpio, D.; Yaghi, O.M. A Porous Covalent Organic Framework with Voided Square Grid Topology for Atmospheric Water Harvesting. *J. Am. Chem. Soc.* **2020**, *142,* 2218−2221.
12. Nguyen, H.; Gropp, C.; Hanikel, N.; Möckel, A.; Lund, A.; Yaghi, O.M. Hydrazine−Hydrazide−Linked Covalent Organic Frameworks for Water Harvesting. *ACS Cent. Sci.* **2022**, *8*, 926−932.
